# Stakeholder-reported perspectives on decentralized clinical trials: A multi-country descriptive survey

**DOI:** 10.1016/j.conctc.2026.101694

**Published:** 2026-09-28

**Authors:** Arghya Biswas

**Affiliations:** School of Clinical Research, Universidad Central de Nicaragua (UCN), Managua, 12104, Nicaragua

**Keywords:** Decentralized clinical trials, Digital health, Patient-centric research, Regulatory barriers, Remote monitoring, Stakeholder perspectives

## Abstract

Decentralized clinical trials (DCTs), which enable trial activities to occur outside traditional clinical trial sites, are reshaping the landscape of clinical research by enabling patient-centric, flexible, and digitally supported trial models. However, widespread adoption faces regulatory, operational, and technological challenges. This study aimed to assess stakeholder perceptions, implementation trends, benefits, and challenges of DCTs across various settings. A cross-sectional, mixed-methods online survey using convenience and snowball recruitment was conducted among 288 stakeholders involved in clinical research, including sponsors, contract research organizations, hospitals/medical research institutions, and technology providers across 38 countries. Findings represent stakeholder-reported experiences rather than population-level adoption. This paper presents quantitative data analyzed using descriptive and exploratory bivariate analyses. Hybrid and full DCT implementation were reported by 144/164 (87.8%) and 63/164 (38.4%) respondents, respectively (independently-reported categories and not mutually exclusive). North America and Asia were the regions most frequently reported by respondents. The most commonly used DCT components were electronic patient-reported outcomes/electronic clinical outcomes assessments [117/161 (72.7%)], remote monitoring [114/161 (70.8%)], and electronic consent [96/161 (59.6%)]. Stakeholders identified key benefits, including improved patient convenience [152/228 (66.7%)] and real-time data collection [136/228 (59.6%)], while key barriers included patient technology literacy [127/228 (55.7%)] and site staff adaptation [123/228 (53.9%)]. Within this sample, hybrid DCTs were selected more frequently on model-effectiveness item; however, the findings do not establish general superiority of one model. Model suitability remains trial-context dependent. Addressing regulatory, infrastructural, and training-related challenges is critical for broader adoption. These findings document stakeholder-reported experience on DCT implementation approaches across diverse healthcare settings.

## Highlights

• This survey assessed stakeholder-reported DCT implementation patterns worldwide.
• The benefits and challenges faced during DCT implementation were also evaluated.
• Hybrid DCTs were frequently implemented and considered more effective.
• The key benefit identified by stakeholders was patient convenience and satisfaction.
• The main challenge identified by stakeholders was patient technology literacy.

## Graphical abstract

**Figure:**
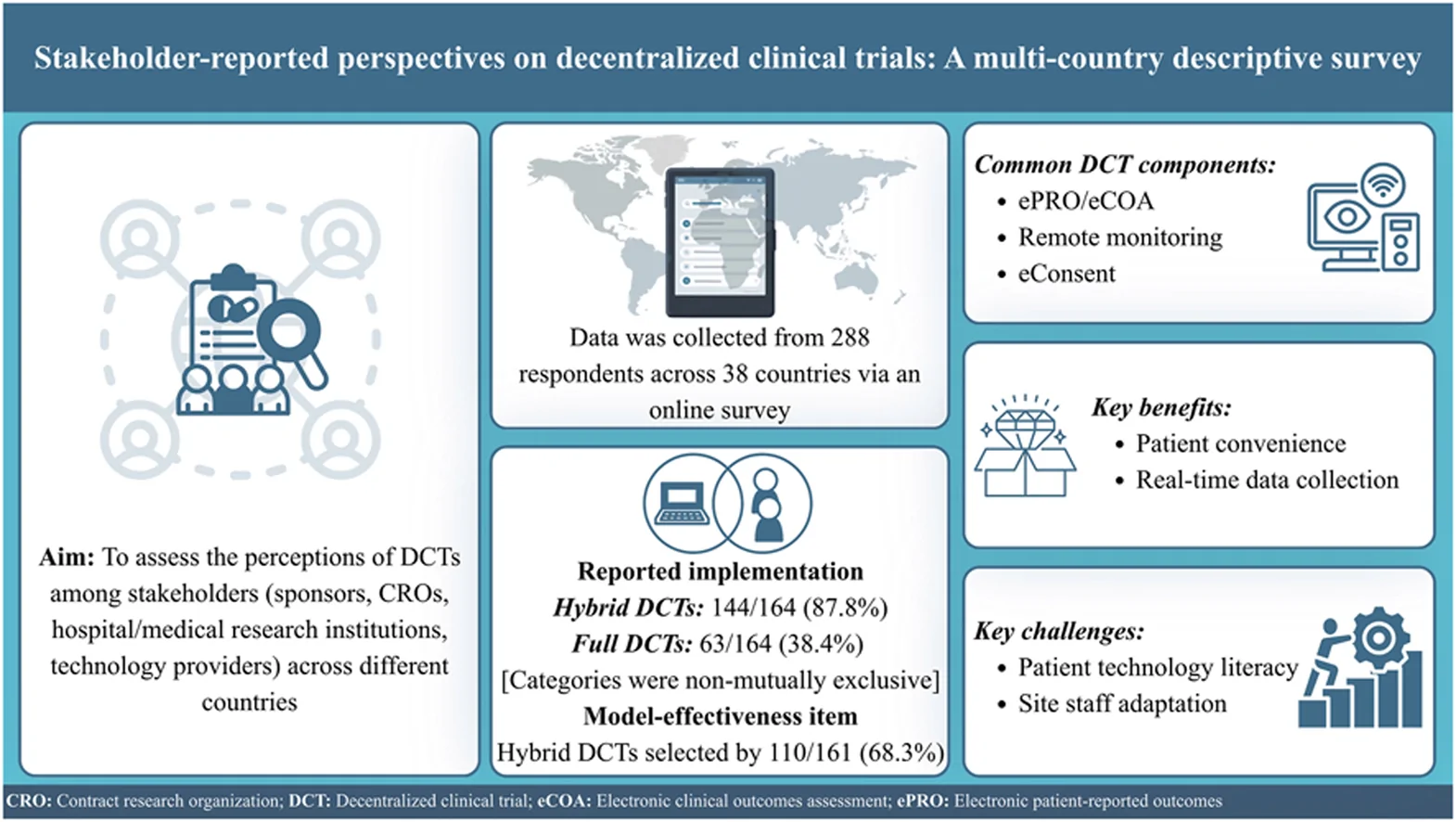

## 1 Introduction

Clinical trials are the cornerstone of evidence generation to establish causal relationships and assess the safety and effectiveness of medical interventions [1]. Globally, the number of clinical trials has surged from 5719 in 1999 to 39 952 in 2024, reflecting a growing emphasis on evidence-based healthcare [2]. Traditional site-based clinical trials often present logistical challenges, including the location of the trial site, the need for frequent visits, and limited time slot availability at the trial site, thereby hindering patient recruitment, delaying trial completion, and increasing operational costs [1,3].

To address these challenges, decentralized clinical trials (DCTs) have emerged as an innovative model. DCTs involve conducting some or all trial-related activities at locations outside of conventional sites, leveraging remote and virtual technologies [4,5]. Full DCTs eliminate the need for conventional site visits by facilitating all trial activities virtually or at more convenient locations, including patients’ residences or local healthcare facilities, while hybrid DCTs combine some trial activities conducted at locations convenient to patients or by using select technologies and solutions with occasional site-based interactions [5,6].

DCTs have existed since the early 2000s [[7], [8], [9]]. The Research on Electronic Monitoring of Overactive Bladder Treatment Experience (REMOTE) trial (2011-2012) was among the first full DCTs conducted under an investigational new drug application, demonstrating outcomes comparable to conventional trials [3,8]. Moreover, the coronavirus disease of 2019 (COVID-19) pandemic further accelerated DCT adoption by rapid integration of telemedicine and remote monitoring to ensure continuity of clinical trials [[7], [8], [9]]. Among trials that continued during the pandemic, 83% used DCT methods [10]. Early pandemic surveys indicated that 76% of pharmaceutical companies, contract research organizations (CROs), and device manufacturers implemented DCTs [11].

Increasing use of digital technologies, including electronic consent (eConsent), virtual consultations, digital data capture, mobile applications, and wearable devices, has further facilitated DCT adoption [12,13]. Artificial intelligence and machine learning may support participant recruitment, site selection, data collection and analysis, and safety monitoring [6]. For instance, a large language model-assisted screening accelerated trial eligibility determination and was associated with more participant enrollments than manual screening in a prospective randomized study embedded within an ongoing heart-failure trial [14]. Newer technologies help reduce duplicate enrollment and fraudulent activities by verifying participants’ eligibility and credibility [6]. DCTs may offer numerous advantages over traditional trials, including faster recruitment, improved participant retention, reduced burden on both participants and sponsors, enhanced diversity in study populations, the ability to capture real-world data, and significant financial benefits for sponsors through improved return on investment [5,6,15].

Regulatory agencies, including the Danish Medicines Agency and Swissmedic, the European Medicines Agency (EMA) [[16], [17], [18], [19]], Clinical Research Malaysia [20], and the United States Food and Drug Administration (USFDA), have published guidance on conducting DCTs [4]. Despite these advancements, DCT adoption remains variable and faces several challenges [15], including coordinating trial activities across diverse nontraditional locations. Effective DCT implementation demands careful planning, collaboration with local healthcare providers and laboratories, rigorous training and monitoring of decentralized trial activities, and ongoing risk assessment and mitigation strategies tailored to nontraditional trial sites [4].

Recent qualitative policy-stakeholder and registry-based studies have identified many of the benefits and challenges associated with DCT implementation [21,22]. The contribution of the present study is therefore not the identification of previously unrecognized themes, but the use of a common survey instrument to quantify and compare the relative salience of established implementation experiences, benefits, and challenges across organization types, seniority levels, and levels of DCT experience within a multi-country stakeholder sample. The findings are descriptive and hypothesis-generating and complement registry-based, qualitative, and policy-level evidence.

Therefore, we conducted an online survey to quantify and compare the current state of DCT adoption across different organizations and regions; analyze perceptions of DCT benefits and challenges based on the type of organizations, stakeholder roles, and level of experience; assess the perceived effectiveness of hybrid versus full DCTs; identify the primary challenges in DCT implementation and its wider adoption; assess the influence of the COVID-19 pandemic on DCT adoption; identify the site perspectives and the perceptions of DCTs among the stakeholders; and find effective strategies to overcome DCT-related challenges. The results provide a descriptive comparison of stakeholder-reported DCT implementation experiences across the settings represented in the sample.

## 2 Methods

### 2.1 Ethics statement

The study involved minimal risk and did not include direct patient participation. However, recognizing the involvement of human participants, the survey protocol was reviewed and approved by the ACE Independent Ethics Committee, Bangalore, India (DCGI Reg. No.: ECR/141/Indt/KA/2013/RR-19). The survey introduction included information on the study's purpose, expected completion time, and assurances of confidentiality and anonymity, and emphasized that participation was entirely voluntary.

### 2.2 Study design

This cross-sectional, mixed-methods study employed a survey-based research design to gather data from various stakeholders in the clinical research ecosystem regarding their perspectives on DCTs. A semi-structured questionnaire was prepared in English and administered online. This paper presents only the quantitative data collected from closed-ended questions.

### 2.3 Respondents and sampling

The study targeted clinical research stakeholders, including sponsors, CROs, technology providers, hospital/trial site staff, and medical research professionals, with prior experience and/or knowledge of DCTs. A non-probability sampling approach combining convenience and snowball techniques was adopted to recruit respondents. The survey link was disseminated via professional networks, email, WhatsApp, and posted on social media platforms such as LinkedIn and Facebook. A formal sample size calculation was not performed as eligible respondents with relevant knowledge and experience were expected to be limited, given that DCT is an emerging topic. However, to account for non-participation and incomplete responses, a minimum target sample size of 250 respondents was set for the predefined one-month data collection period. The final sample comprised 288 respondents from 38 countries across Asia, Africa, Australia, Europe, the Middle East, North America, and South America. Country-wise response counts were uneven and insufficient to support country-level comparisons or inferences. Country labels indicate respondent location only and should not be interpreted as representative of national DCT activity, adoption rates, or regulatory maturity.

Respondents' knowledge of DCTs was assessed using a structured questionnaire administered, and those self-reporting ‘No knowledge’ of DCTs were excluded from the analysis (n = 24).

Respondents were not requested to confirm that they were authorized to share opinions, as they did not disclose their organizations. No monetary or non-monetary incentives were offered for participation. Duplicate entries were screened by matching respondent name and email ID, and in such cases, only the first complete response from each individual was retained for analysis.

This quantitative descriptive survey documents stakeholder-reported perspectives on DCT implementation. The convenience and snowball sampling via social media means findings represent stakeholder-reported implementation patterns rather than population-level prevalence estimates. Patient perspectives were assessed indirectly through stakeholder-reported impressions, and technology elements were analyzed as aggregate components. Readers should interpret results as descriptive stakeholder perspectives from engaged early-adopter professionals, not as market prevalence data or technology-specific implementation guidance.

### 2.4 Questionnaire design and data collection

A semi-structured questionnaire with 22 open- and closed-ended questions, including four subparts in one of the questions, was developed based on a review of key peer-reviewed DCT studies and major organization reports on DCT implementation [[23], [24], [25], [26], [27]], stakeholder perspectives, and regulatory considerations, together with input from clinical research experts experienced in DCTs. This questionnaire was pilot-tested among nine eligible respondents from Denmark and India to gather feedback to ensure clarity and feasibility. Modifications were made based on the pilot-test results to improve the flow of questions and eliminate redundancies. The pilot testing was conducted in Denmark and India to ensure representation from both a mature DCT environment (Europe) and an emerging DCT market (Asia), allowing us to assess clarity and feasibility across diverse regulatory and operational contexts.

Following the modifications guided by respondents’ feedback and expert consultation, the final questionnaire included 17 questions. Data were collected using this finalized semi-structured, self-administered, online questionnaire. It was organized into five sections: (1) respondent demographics and experience, (2) DCT implementation and characteristics, (3) perceived benefits and challenges, (4) patient and site perspectives, and (5) future outlook. The questionnaire included both closed-ended (single- and multiple-response) and open-ended questions. The closed-ended questions included in the survey are listed in Fig. 1.

**Fig. 1:**
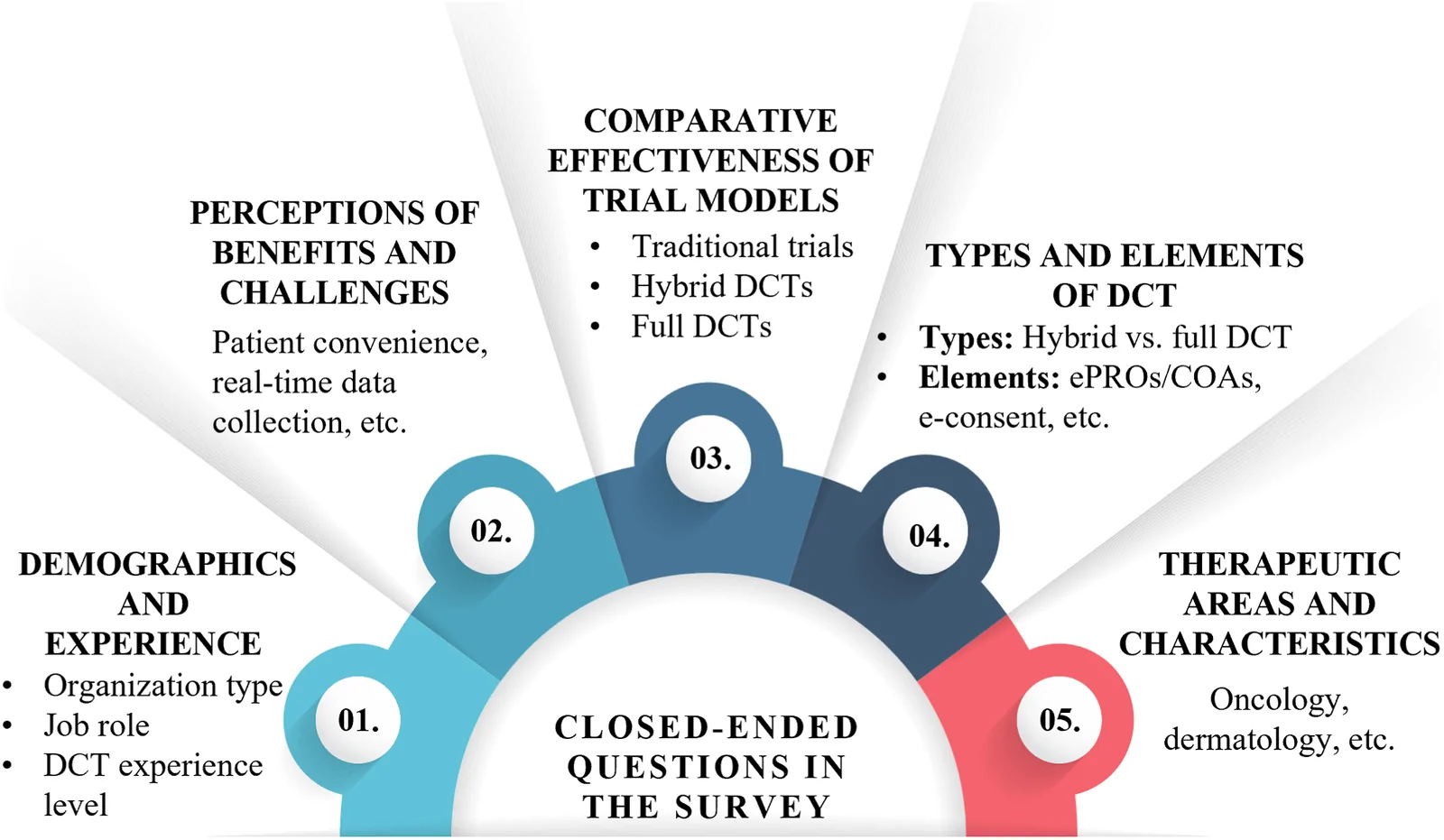
Closed-ended questions included in the survey **DCT:** Decentralized clinical trial; **eCOA:** electronic clinical outcomes assessments; **ePROs:** electronic patient-reported outcomes.

The survey introduction provided respondents with standardized operational definitions, defining a ‘Full DCT’ as a clinical trial where all study visits and assessments are conducted remotely, without physical site visits, ‘Hybrid DCT’ as a clinical trial combining traditional site-based elements with remote or virtual components (DCT elements), and ‘DCT Elements’ as ‘components of a clinical trial that enable remote or off-site data collection and participant interaction,’ which guided respondents toward self-classification and reporting (Supplementary Information File S1). The survey did not impose a numerical threshold for the proportion of decentralized activities; rather, hybrid DCTs retained traditional site-based elements, whereas full DCTs had no physical site visits under the survey definition.

Survey routing was based on respondents’ experience and organizational implementation. Respondents who selected “Familiar with the concept of DCTs, no direct experience” were routed to Question 10, and those selecting “No knowledge of DCTs” ended the survey. Respondents reporting that their organization had not implemented a DCT were also routed to Question 10. Consequently, Question 9, which asked *“In your experience, which approach has been most effective?”*, was answered only by respondents who reached the experience and implementation module; subsequent perception items could include DCT-familiar respondents without direct experience. Each respondent could submit only one response through a unique survey link, and IP address tracking was used to minimize duplicate submissions.

The final questionnaire was hosted on SurveyMonkey and is provided in the Supplementary Information File S1. The data collection was carried out from 6 August 2024 to 9 September 2024.

### 2.5 Data analysis

Quantitative data were coded in Microsoft Excel and analyzed using IBM SPSS Statistics version 20. Univariate analysis (frequencies, percentages, and weighted means) was conducted for descriptive statistics. Statistical testing was exploratory and descriptive in nature and was not intended to infer population-level effects or causal relationships. It was performed using Chi-square and other bivariate analyses at a 95% confidence interval (CI) with significance set at p < 0.05. For the benefit/challenge items, “benefit” combined moderate and significant benefit responses, whereas “challenge” combined moderate and significant challenge responses. The percentages for these items used a fixed denominator, which included all respondents who entered the benefit/challenge module, with “No difference,” “Can't say/Not sure,” and item nonresponse not included in the corresponding numerators. Results were cross-tabulated by organization type, seniority level, and experience level to examine variations in stakeholder perceptions across these respondent groups.

## 3 Results

### 3.1 Stakeholder demographics and experience with decentralized clinical trials

A total of 288 stakeholders from 38 countries participated in the survey. The number of respondents from each country is provided in Supplementary Information File S2. The final analytical cohort comprised 264 respondents after application of the prespecified knowledge criterion described in the Methods. The demographic and professional characteristics of the respondents are summarized in Table 1.

**Table 1:** Respondent characteristics (N = 288).

Characteristics	Number	Percentage (%)
Organization Type		
Sponsor pharmaceutical/biotech company	121	42.0
Hospital/Medical research institutions	84	29.2
Contract research organizations/technology providers	74	25.7
Others*	9	3.1
***Designation***		
Executive/Senior Management (e.g., Vice President, Director, Principal Investigator)	114	39.6
Associate/Operational staff (e.g., Executive Project Manager, Study Coordinator)	93	32.3
Middle management (e.g., Manager, Senior Manager, Sub-Investigator)	79	27.4
Others^#^	2	0.7
***Experience with DCTs***		
Worked on DCT in the current organization	95	33.0
Worked on DCTs	58	20.2
The current organization has conducted DCT	30	10.4
Familiar with the concept of DCTs without direct experience	81	28.1
No knowledge of DCTs^†^	24	8.3

**DCT**: Decentralized clinical trial; **N:** Total number of respondents; *: Consultants (n = 3), nonprofit organizations (n = 1), partner organizations (n = 1), directors (n = 1), pre-competitive cross-industry consortia (n = 1), patient advocacy groups (n = 1), and publications (n = 1); #: Retired senior professional, Subject Matter Expert/Senior consultant; ^†:^ Excluded from DCT outcome analyses.

### 3.2 Adoption patterns and characteristics of decentralized clinical trials

Hybrid DCTs were more commonly reported as being implemented [144/164 (87.8%)] than full DCTs [63/164 (38.4%)] across the respondents’ current organizations. These percentages reflect independently reported deployment rates and are not mutually exclusive.

As shown in Table 2, North America was the most frequently reported region in which respondents’ organizations had deployed DCT elements [92/161 (57.1%)], followed by Asia [80/161 (49.7%)] and Europe [79/161 (49.1%)]. Within each reported region, involvement in hybrid DCTs was reported more frequently than in full DCTs.

**Table 2:** Percentage of respondents deploying decentralized clinical trial elements across regions (N = 161).

Regions	Number [percentage (%)] of respondents
Africa	18 (11.2%)
Middle East	20 (12.4%)
Australia/Oceania	31 (19.3%)
South America	36 (22.4%)
Europe	79 (49.1%)
Asia	80 (49.7%)
North America	92 (57.1%)

**DCT:** Decentralized clinical trial; **N:** Total number of respondents.

The most frequently deployed DCT elements reported by the respondents included electronic patient-reported outcomes/electronic clinical outcomes assessments [117/161 (72.7%)], remote monitoring [114/161 (70.8%)], eConsent [96/161 (59.6%)], and direct-to-patient shipping [87/161 (54.0%)], as shown in Table 3.

**Table 3:** Percentage of respondents deploying various decentralized clinical trial elements (N = 161).

Deployment of DCT elements	Number [percentage (%)] of respondents
ePRO/eCOA	117 (72.7%)
Remote monitoring	114 (70.8%)
eConsent	96 (59.6%)
Direct-to-patient shipping	87 (54.0%)
Home health visits	78 (48.4%)
Digital patient engagement tools	77 (47.8%)
Telemedicine	72 (44.7%)
Digital recruitment	71 (44.1%)
Wearable/sensors	70 (43.5%)
Flexible sample collection	57 (35.4%)
Virtual clinical trial site/metasite	55 (34.2%)
Remote adverse event collection	53 (32.9%)
Others*	2 (1.2%)

**DCT:** Decentralized clinical trial; **eConsent:** electronic consent; **ePRO/eCOA:** electronic patient-reported outcome/electronic clinical outcomes assessments; **N:** Total number of respondents; *: Dose guidance software & community studies.

Table 4 displays DCT deployment by therapeutic area. Key therapeutic areas for DCT deployment included cardiovascular and metabolic disorders [61/161 (37.9% each)], oncology [60/161 (37.3%)], and immunology/infectious diseases [58/161 (36.0%)].

**Table 4:** Deployment of decentralized clinical trials elements by therapeutic area (N = 161).

Therapeutic area of DCTs	Number [percentage (%)] of respondents
Cardiovascular	61 (37.9%)
Metabolic disorders	61 (37.9%)
Oncology	60 (37.3%)
Immunology/infectious disease	58 (36.0%)
Respiratory disease	43 (26.7%)
Rare diseases	39 (24.2%)
Neurology	39 (24.2%)
Dermatology	26 (16.1%)
Rheumatology	15 (9.3%)
Others	21 (13.0%)

**DCT:** Decentralized clinical trial; **N:** Total number of respondents; **Note:** Other includes ophthalmology, gastroenterology, exercise physiology and rehabilitation, insomnia, cancer screening, nutraceuticals, aging in place, internal medicine, cell and gene therapies, optometry, women's health (menopause), mental health, diagnostic, diabetic neuropathic pain, digital health, and diagnostic trials.

As presented in Fig. 2, most DCTs were conducted in Phase III [122/161 (75.8%)] and Phase II [77/161 (47.8%)], with trial durations typically ranging from one to two years [80/161 (49.7%)] or more than two years [71/161 (44.1%)]. Over half of the respondents [83/161 (51.6%)] reported experience with DCTs, which were global trials. Post-COVID-19 pandemic, 111 of 161 (69.0%) stakeholders reported observing an increased DCT adoption.

**Fig. 2:**
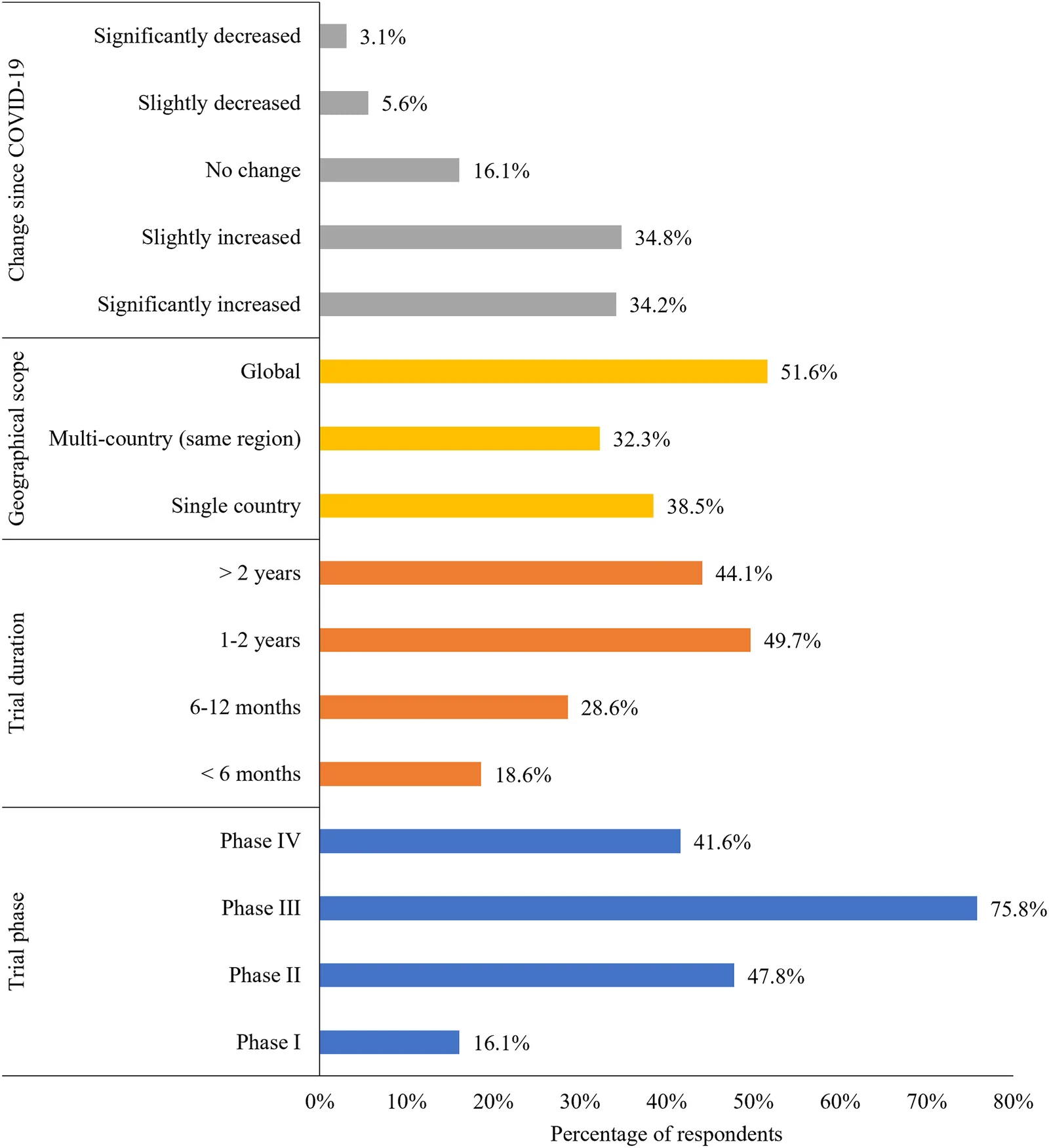
Respondent-reported characteristics of decentralized clinical trials (N = 161) **COVID-19:** Coronavirus disease 2019.

### 3.3 Stakeholder-reported organizational implementation and perceived model effectiveness

Fig. 3A presents respondent-reported organizational implementation of hybrid DCTs by organization type. Hybrid DCT implementation was reported by 47 of 49 (95.9%) respondents from sponsor pharmaceutical/biotechnology companies, 37 of 41 (90.2%) from CROs/technology providers, and 22 of 26 (84.6%) among hospitals/medical research institutions. These percentages describe reported organizational implementation and not respondent preferences.

**Fig. 3:**
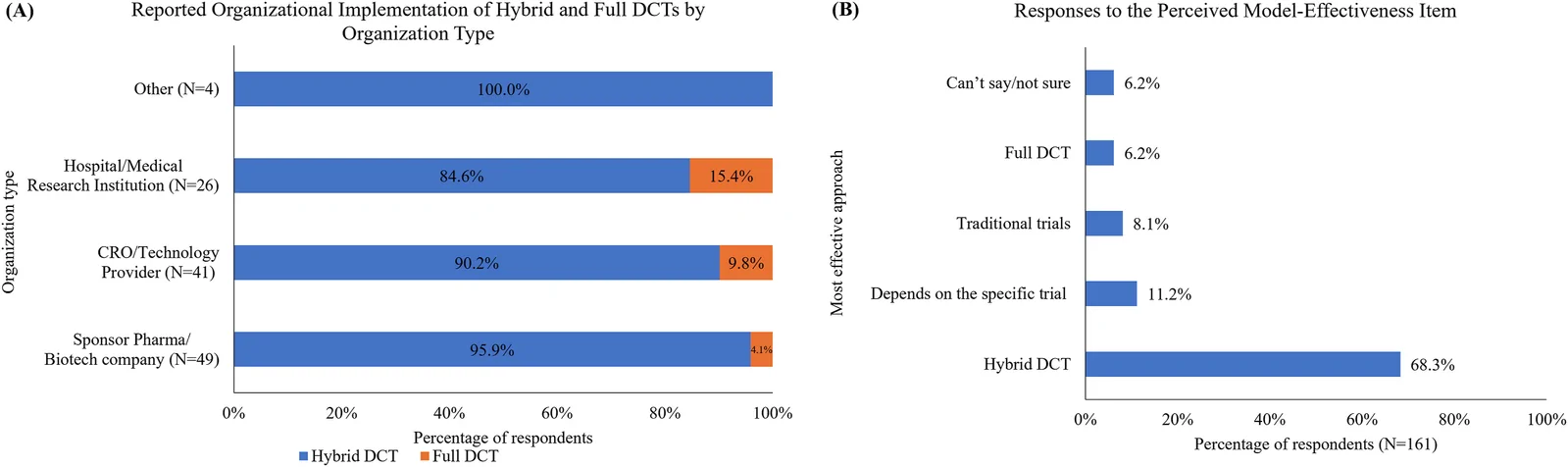
Stakeholder-reported organizational implementation and perceived model effectiveness: (A) implementation of hybrid and full DCTs by organization type; (B) responses to the model-effectiveness item **CRO:** Contract research organization; **DCTs:** Decentralized clinical trials; **N:** Total number of respondents.

Among the 161 respondents who answered the question, *“In your experience, which approach has been most effective?”*, 110 (68.3%) selected hybrid DCTs, 10 (6.2%) selected full DCTs, 13 (8.1%) selected traditional clinical trials, and 18 (11.2%) indicated that the most effective model depends on the specific trial context, and 10 (6.2%) selected “Can't say/Not sure” (Fig. 3B).

### 3.4 Stakeholder perceptions of concerns and barriers in decentralized clinical trials

The primary concern of clinical trial sites regarding DCTs experienced by the majority of respondents was technology challenges [77/107 (72.0%)]. This was followed by data quality and integrity issues [52/107 (48.6%)], changes in staff roles and responsibilities [45/107 (42.1%)], patient safety monitoring [45/107 (42.1%)], and regulatory compliance uncertainties [38/107 (35.5%)]. Other notable concerns included loss of control over trial processes [37/107 (34.6%)], reduced site revenue [36/107 (33.6%)], loss of patient engagement and relationships [31/107 (29.0%)], lack of involvement of sites in trial planning [29/107 (27.1%)], and concerns about long-term relevance of traditional site roles [19/107 (17.8%)]. Additional concerns were cited by 9 of 107 (8.4%) respondents and categorized as ‘Other’.

As presented in Table 5, which outlines stakeholder perceptions of barriers to the wider adoption of DCTs, regulatory uncertainty and resistance to change (internal or external) were reported as the primary obstacles, each with a mode rank of one. Other barriers, such as high implementation costs, received high weighted mean ranks. Several barriers (e.g., patient preference for traditional trials and inability to measure return on investment) received similar weighted mean and mode ranks.

**Table 5:** Stakeholder perceptions of barriers to wider adoption of decentralized clinical trials.

Primary Barrier	Weighted Mean Rank	Mode Rank
Regulatory uncertainty	5.0	1
Resistance to change (internal/external)	4.7	1
Operational and logistical challenges	4.7	3
High implementation costs	5.7	3
Lack of standardization across industry	5.0	4
Technology limitations	5.2	4
Site adoption	5.7	5
Concerns about data quality/data privacy	5.6	9
Patient preference for traditional trials	6.6	10
Inability to measure return on investment	6.8	10

Total respondents	183	

### 3.5 Stakeholders’ perceptions of benefits and challenges of decentralized clinical trials

Stakeholders highlighted a range of quantifiable benefits of DCTs over traditional trials. For questions allowing multiple responses, participants could select more than one option; percentages for each category were calculated independently and were not expected to sum to 100%. A total of 228 respondents entered the benefit/challenge module. As depicted in Fig. 4, improved patient convenience emerged as the top advantage, cited by 152/228 (66.7%) respondents, followed closely by real-time data collection [136/228 (59.6%)], better patient retention [129/228 (56.6%)], increased patient diversity [124/228 (54.4%)], and enhanced recruitment [121/228 (53.1%)]. In addition, convenience was the most beneficial aspect of DCTs reported directly by patients in the DCTs in which the respondents worked. Other positive aspects of DCTs reported directly by patients included reduced travel time, better integration with daily life, improved communication with site staff, and access to real-time health data.

**Fig. 4:**
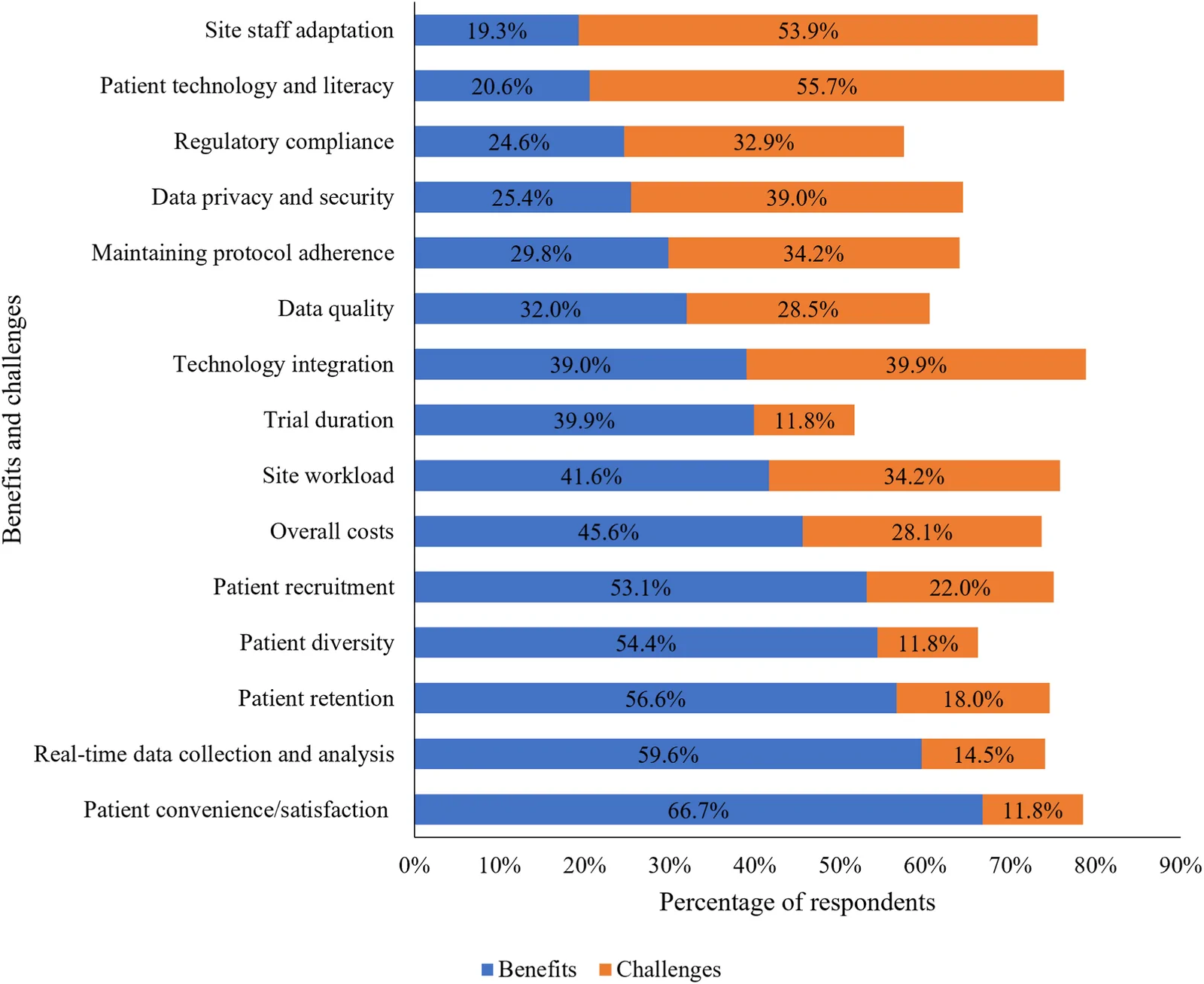
Perception of respondents about the benefits and challenges of decentralized clinical trials (N = 228) **DCT:** Decentralized clinical trial.

On the other hand, prominent challenges were also numerically evident, and patient technology literacy was flagged by 127/228 (55.7%), site staff adaptation by 123/228 (53.9%), followed by technology integration issues [91/228 (39.9%)], data privacy concerns [89/228 (39.0%)], and difficulty maintaining protocol adherence [78/228 (34.2%)].

The perceptions of the benefits and challenges of DCTs varied depending on stakeholders' organization type, role, and prior experience. To better understand these differences, an analysis was stratified based on the stakeholders’ background.

#### 3.5.1 Perception of benefits based on stakeholder characteristics

The following are the benefits of DCTs as reported by the stakeholders and categorized based on the:

• **Organization Type:** Patient convenience/satisfaction was perceived as the most beneficial aspect of DCTs by 79.5% (62/78) of stakeholders from sponsor pharmaceutical/biotech companies, 83.9% (47/56) from CROs/technology providers, and 73.1% (38/52) from hospital/medical research institutions.
• **Seniority level:** The most beneficial aspect of DCTs was identified as patient convenience and satisfaction by 82.3% (65/79) of stakeholders holding executive, senior management roles, 77.4% (41/53) holding middle management roles, and 78.0% (46/59) of associate/operational roles.
• **Experience level:** Patient convenience/satisfaction was perceived as the most beneficial aspect by 75.0% (30/40) of stakeholders with DCT experience, 88.2% (15/17) of stakeholders whose organizations conducted DCTs, and 80.3% (49/61) of stakeholders who were familiar with the concept of DCTs but had no direct experience. Notably, 80.8% (59/73) of stakeholders who worked on DCTs and their organization conducted DCTs considered real-time data collection and analysis as the key benefit.

#### 3.5.2 Perception of challenges based on stakeholder characteristics

The following are the challenges of DCTs as reported by the stakeholders and categorized based on the:

• **Organization Type:** Site staff adaptation was considered the most challenging aspect of DCTs by 71.1% (59/83) of stakeholders from sponsor pharmaceutical/biotech companies and 72.7% (40/55) from CROs/technology providers. Meanwhile, 65.4% (34/52) of stakeholders from hospital/medical research institutions identified patient technology literacy as the key challenge.
• **Seniority level:** Site staff adaptation was viewed as the main challenge by 68.4% (54/79) of stakeholders holding executive, senior management roles, whereas 71.2% (37/52) holding middle management roles, and 62.3% (38/61) of associate/operational roles considered patient technology literacy as the primary challenge.
• **Experience level:** Patient technology literacy was perceived as the most challenging aspect by 62.5% (25/40) of stakeholders with DCT experience, 64.7% (11/17) of stakeholders whose organizations conducted DCTs, and 73.0% (46/63) of stakeholders who were familiar with the concept of DCTs but had no direct experience. However, 65.3% (47/72) of stakeholders who worked on DCTs and their organization conducted DCTs considered site staff adaptation as the key challenge.

### 3.6 Stakeholder strategies to address decentralized clinical trial implementation challenges

To explore actionable solutions for overcoming challenges in the implementation of DCTs, respondents were asked to rank six commonly recommended approaches. As demonstrated in Table 6, conducting pilot studies before full-scale implementation received a mode rank of one and a weighted mean rank of 3.2. Partnering with experienced technology providers and developing comprehensive staff training programs received a weighted mean rank of 3.0 each and a mode rank of two and four, respectively.

**Table 6:** Effective strategies to overcome the possible challenges with decentralized clinical trials.

Strategies for overcoming the possible challenges	Weighted mean rank	Mode rank
Partnering with experienced technology providers	3.0	2
Developing comprehensive staff training programs	3.0	4
Early engagement with regulatory bodies	3.6	5
Implementing robust data management	4.2	5
Additional support for patient engagement	4.0	6
Conducting pilot studies before full implementation	3.2	1

**Total respondents**	**203**	

## 4 Discussion

This study describes stakeholder-reported DCT implementation patterns and perceived benefits and challenges of DCTs among respondents across 38 countries. In the overall analytical sample, hybrid DCT implementation was reported more frequently than full DCT implementation (87.8% vs. 38.4%). Among the 161 respondents who answered the model-effectiveness item, 68.3% selected hybrid DCTs, 6.2% selected full DCTs, 8.1% selected traditional clinical trials, 11.2% selected the context-dependent option, and 6.2% selected “Can't say/Not sure.” Stakeholders highlighted several patient-centric advantages of DCTs, with the most prominent being improved patient convenience (66.7%), real-time data collection (59.6%), better patient retention (56.6%), increased diversity (54.4%), and enhanced recruitment (53.1%).

The most cited challenges by stakeholders that continue to hinder broader DCT adoption were patient technology literacy, site staff adaptation, and technology integration. Data privacy and protocol adherence were also notable concerns. Data privacy concerns were reported by 39.0% of respondents and remain an important ethical consideration.

The more frequent selection of hybrid DCTs in this sample may reflect the frequent stakeholder-reported organizational implementation of hybrid approaches and the operational flexibility they offered by combining the benefits of remote technologies and in-person touchpoints. This observation aligns with recent literature by Barge R. et al., which reported that about half of the sites and patients expressed comfort with hybrid DCTs. Both patients and sites expressed preferences for hybrid and full DCTs over conventional trials [28]. Hybrid and full DCTs should, however, be viewed as complementary options along a continuum rather than as competing models. Hybrid designs may be more appropriate when site-based procedures, investigational-product administration, imaging, invasive assessments, or intensive safety monitoring are required, whereas full DCTs may be feasible when visits and assessments can be conducted remotely while maintaining participant safety and data quality. Model selection should therefore be guided by the research question, participant population, intervention, endpoint requirements, safety considerations, and operational context [4,16,29]. While DCTs share many core principles with traditional trials, their implementation requires distinct approaches. Since DCTs utilize remote technologies, data flows are largely digital and continuous, necessitating secure integration and real-time monitoring. Regulatory and ethical processes must accommodate remote consent, data privacy, maintaining patients’ safety, and coordination across diverse locations. Patient engagement strategies in DCTs are more personalized and flexible, emphasizing convenience, adherence, and retention. These factors demand tailored planning, training, and oversight [[4], [5], [6],^13^].

Given the complexity of DCT operations, early and sustained engagement of sponsors, investigators, site personnel, trial monitors, and management teams is essential during trial design [30]. Recognizing this, initiatives such as the Clinical Trials Transformation Initiative (CTTI) have launched multi-stakeholder projects addressing the legal, regulatory, and operational barriers to DCTs in the United States [15]. Similarly, Coert RMH. et al. identified key barriers to DCT adoption in Europe, including regulatory acceptance, technical challenges, and limited stakeholder knowledge. Conversely, factors like stakeholder involvement, pilot study exposure, and clear patient support mechanisms were found to facilitate successful DCT implementation [31].

The patient-centric advantages reported in this study align with studies by Chodankar D. et al. and Sommer C. et al., which emphasized expanded access, improved patient retention, convenience, and satisfaction facilitated by DCTs [32,33]. The technology-related challenges are also consistent with previous research [13,34], underscoring the need for intuitive, secure, and interoperable digital platforms supported by comprehensive training. A practical example is provided by the fully decentralized CHIEF-HF trial. Although 18 sites contacted 130 832 potential participants, only 2.0% entered the web-based recruitment pathway, and recruitment yield varied substantially across sites. At the highest-enrolling site, electronic medical record portal messaging produced a higher opt-in rate than email alone (7.8% versus 4.4%), illustrating the potential value of locally integrated, multichannel recruitment strategies rather than reliance on a single digital channel [35].

Additionally, operational experience from the pan-European RADIAL proof-of-concept trial illustrates these challenges in practice. The trial compared conventional, hybrid, and fully decentralized approaches across six European countries and required coordination of multiple technologies, clinical sites, vendors, and participant-facing processes. Implementation strategies included phased technology deployment, risk-based validation, structured site and participant onboarding, role-specific training, issue-tracking systems, and access to ongoing human technical support. These experiences demonstrate that successful decentralization requires not only suitable digital tools but also coordinated workflows, stakeholder preparation, and continuing support [36,37]. As the benefits and barriers reported in the survey are already well recognized in the literature, the contribution of this study lies in applying a common survey instrument to compare how frequently they were reported across organization types, seniority levels, and levels of DCT experience within this multi-country sample.

Guidance from the USFDA and EMA emphasizes patient safety and data integrity when implementing DCT components. While both agencies highlight risk-based monitoring, robust communication, and action plans, there are nuanced differences [16]. The International Council for Harmonization of Technical Requirements for Pharmaceuticals for Human Use (ICH) guideline for good clinical practice (GCP) E6(R3) emphasizes flexibility, risk-based quality management, and integration of digital technologies to enhance trial efficiency and participant engagement. It clarifies stakeholder roles and responsibilities to manage DCT elements effectively. The ICH E6(R3) Annex 2, endorsed on 03 June 2026 and incorporated into the ICH E6(R3) guideline on 16 June 2026, provides additional GCP considerations and examples of trials incorporating DCT components [29,38]. Similarly, uncertainty and resistance to change (internal or external), which emerged as the top perceived barriers to wider DCT adoption, highlight the need for clear, harmonized, and widely accepted operational standards to support multi-regional implementation, aligning with the recommendations of Halle C. et al. [39].

Although this study does not provide prescriptive guidance on selecting specific DCT technologies or models, it offers a stakeholder-reported comparison of implementation experiences and perceived benefits and challenges across the groups represented in the sample. The more frequent selection of hybrid DCTs should not be interpreted as evidence that one model is universally superior or that organizations should progress from hybrid to full DCTs. Rather, decentralized elements should be selected according to trial-specific, participant, safety, and operational requirements.

This study has a few limitations. The survey was prepared in English, assuming respondents, being working professionals, would have sufficient proficiency. However, differences in responses between native and non-native English speakers were not assessed, which may have introduced bias. As the survey link was disseminated online, we could not reliably estimate participation and response rates, limiting the assessment of potential non-response bias. Also, online social media recruitment limits identity verification without institutional credentials or in-person confirmation, which is an inherent limitation. We used a convenience and snowball sampling strategy without predetermined quotas or response rate tracking, which limits statistical generalizability and may have introduced selection bias, including overrepresentation of professionals engaged in DCTs. Accordingly, the findings reflect the views of this self-selected sample and should not be generalized to country-level, regional, organizational, or market-level DCT adoption.

The operational distinction between full and hybrid DCTs simplified a heterogeneous continuum of decentralized trial designs into two survey categories. DCTs were intentionally defined broadly in this study to reflect real-world practice, where DCTs are implemented as heterogeneous combinations of technologies and operational components rather than a standardized model. Consequently, individual DCT technologies contribute asymmetrically to trial execution, benefits, and challenges, but we did not attempt to quantify the independent effect or relative contribution of each.

The benefits and challenges analyses included 61 and 63 respondents, respectively, who reported familiarity with DCTs but no direct DCT experience. Their responses may therefore reflect professional awareness or organizational exposure rather than first-hand operational experience, which may have influenced the perceptions reported.

Regional percentages and findings represent respondent-reported implementation experiences, not population-level adoption rates. The respondents distributed across 38 countries and multiple therapeutic areas may skew toward early adopters and technology-engaged stakeholders.

Country-specific interpretations should be avoided, as the number of respondents per country varied and was insufficient for national-level comparisons. The country distribution reflects respondents’ location rather than representative DCT adoption within those countries. Also, patient perspectives were captured indirectly through stakeholder reports rather than being obtained directly from patients. Therefore, readers should interpret results as second-hand perspectives from direct implementation rather than patient-reported evidence.

## 5 Conclusion

Within this self-selected stakeholder sample, hybrid DCTs were reported as implemented more frequently and were selected more often on the model-effectiveness item than full DCTs. Respondents commonly identified patient convenience and real-time data collection as benefits, while patient technology literacy and site staff adaptation were commonly identified as challenges. These findings are descriptive rather than population-level estimates, and the choice between hybrid and full DCTs should be based on the trial context and its scientific, participant, safety, and operational requirements.

## 6 Future directions

To support the sustainable scaling of DCTs, future initiatives should prioritize:

• **Capacity-building and training**: Enhancing digital literacy among patients and building adaptability in site staff through structured training programs.
• **Regulatory harmonization**: Aligning international guidance to reduce ambiguity and streamline multi-regional trial approvals.
• **Infrastructure development**: Investing in interoperable digital platforms and remote monitoring tools to ensure consistency and efficiency.
• **Operational readiness**: Encouraging the use of pilot studies, particularly in underrepresented therapeutic areas and geographies, to identify and address implementation gaps.
• **Impact and profiles of individual technologies**: Disaggregating DCT components and evaluating the differential impact, feasibility, and cost-benefit profiles of individual technologies across trial phases, therapeutic areas, and patient populations.
• **Strategic partnerships**: Collaborating with experienced technology providers to strengthen technical execution and data integrity.
• **Tailored stakeholder strategies**: Developing targeted interventions for specific settings, such as hospitals or community-based sites, to support broader adoption.

Future studies should evaluate the feasibility and performance of specific decentralized elements in defined trial contexts. Additionally, further research is warranted to explore direct patient perspectives across diverse regions and populations, ideally through a dedicated global patient survey. Such insights will be instrumental in shaping truly inclusive and effective DCT frameworks.

## Funding

This research did not receive any specific grant from funding agencies in the public, commercial, or not-for-profit sectors.

## Declaration of competing interest

The authors declare that they have no known competing financial interests or personal relationships that could have appeared to influence the work reported in this paper.

## Data Availability

Data will be made available on request.

## Appendix A Supplementary data

**S1 File** Questionnaire. The structured survey questionnaire used to collect data from respondents.Multimedia componet 1

**S2 File** Country-Wise Response Distribution.Multimedia componet 2
