# Supplementary material for: Stakeholder-reported perspectives on decentralized clinical trials: A multi-country descriptive survey
Source: Contemp Clin Trials Commun. 2026 Sep 28;54:101694. doi: 10.1016/j.conctc.2026.101694 (PMC13657102; doi:10.1016/j.conctc.2026.101694)
Supplement: Supplementary file 1 [file mmc1.pdf]

ANNEXURE I – Questionnaire

1. WELCOME&INTRODUCTION

Dear Participant,

I invite you to participate in this important survey on Decentralized Clinical Trials (DCTs) for my academic research. Your insights are crucial in understanding the current landscape, challenges, and future directions of DCTs in the pharmaceutical industry.

Key Definitions:
• **Full DCT:** A clinical trial where all study visits and assessments are conducted remotely, without physical site visits.
• **Hybrid DCT:** A clinical trial combining traditional site-based elements with remote or virtual components (DCT elements).
• **DCT Elements:** Components of a clinical trial that enable remote or off-site data collection and participant interaction.

The survey will take approx.15 minutes and your responses will be kept strictly confidential, anonymized, and used solely for academic research purposes, in compliance with data protection laws.

Your participation is vital in shaping strategies to overcome challenges in DCT adoption. As a thank you, I'll share the survey results with all participants, providing valuable insights into industry trends.

To begin, please click

'Next'. Best regards,
Arghya Biswas
Email:biswas.arghya@gmail.com

2. DEMOGRAPHICS AND EXPERIENCE

* 1. What best describes your organization?

1 Sponsor Pharma /Biotech Company

2 Contract Research Organization (CRO)/Technology Provider

3 Hospital/Medical Research Institution

4 Other (please specify)

* 2. What best describes your level of seniority?

1 Executive/Senior Management (e.g., Vice President, Director, Principal Investigator)
2 Middle Management (e.g., Manager, Senior Manager, Sub-Investigator)
3 Associate/Operational Staff (e.g., Executive, Project Manager, Study Coordinator)
4 Other (please specify)

* 3. What is your experience with DCTs?

1 I've worked on DCTs

2 My organization has conducted DCTs

3 Both1and2

4 Familiar with concept of DCTs, no direct experience……..**Skip to Qs. 10**

5 No knowledge of DCTs**----STOP (END OF SURVEY)**

3. DCT IMPLEMENTATION AND CHARACTERISTICS

* 4. Which type of DCTs has your organization implemented?

1 Full DCT

2 Hybrid DCT

3 No DCT implemented……..**Skip to Qs 10**

* 5. In which regions has your organization deployed DCT elements? (Select all that applies)

1.North America

2.South America

3.Europe

4.Middle East

5.Africa

6.India

7.Asia (excluding India)

8.Australia/Oceania

9.Can't Say/Not Sure

* 6.Which DCT elements have your organization deployed?(Please select all that apply)

1. Virtual clinical trial site /Metasite
2. Digital Recruitment
3. eConsent
4. Telemedicine
5. ePRO/ eCOA
6. Wearable/sensors(DHT)
7. Digital Patient engagement tools
8. Remote adverse Event Collection
9. Direct-to-patient shipping
10. Home Health Visits
11. Flexible Sample Collection
12. Remote monitoring
13. Other (please specify)

14. Can't Say/Not Sure

* 7. In which therapeutic area(s) has your organization implemented DCT elements? (Please select all that applies)

1. Cardiovascular
2. Dermatology
3. Immunology/Infectious diseases
4. Metabolic disorders
5. Neurology
6. Oncology
7. Respiratory diseases
8. Rare diseases
9. Rheumatology
10. Other(please specify)

11. Can't Say/Not Sure

* 8. What are the characteristics of the DCTs your organization has conducted?

8.1 Trial phases (Select Below, all that applies)

1. Phase I
2. Phase II
3. Phase Ill
4. Phase IV
5. Can't Say/Not Sure

8.2 Typical Trial duration (Select Below, all that applies)

1. Less than 6 months
2. 6-12 months
3. 1-2 years
4. More than 2 years
5. Can't Say/Not Sure

8.3 Geographic scope (Select Below, all that applies)

1. Single Country
2. Multi-country(same region)
3. Global
4. Can't Say/Not Sure

8.4 Change since C0VID-19 pandemic (Select Below, only one)

1. Significantly increased
2. Slightly increased
3. No Change
4. Slightly decreased
5. Significantly decreased

* 9. In your experience, which approach has been most effective?

1. Full DCT
2. Hybrid DCT
3. Traditional trials
4. Depends on the specific trial (please explain)
5. Can’t say/Not sure

4. BENEFITS, IMPACTS AND CHALLENGES OF DCTs

*10.For each of the following aspects, indicate whether DCTs present a **Benefit** or a **Challenge** compared to traditional clinical trials? (Please answer all the below statements)

	Significant challenge (1)	Moderate challenge (2)	No difference (3)	Moderate benefit (4)	Significant benefit (5)	Can't Say/Not Sure
1. Patient recruitment	0	0	0	0	0	
2. Patient retention	0	0	0	0	0	
3. Trial duration	0	0	0	0	0	
4. Overall costs	0	0	0	0	0	
5. Data quality	0	0	0	0	0	
6. Patient diversity	0	0	0	0	0	
7. Patient convenience / satisfaction	0	0	0	0	0	
8. Site workload	0	0	0	0	0	
9. Real-time data collection & analysis	0	0	0	0	0	
10. Regulatory compliance	0	0	0	0	0	
11. Technology integration						
12. Data privacy & security						
13. Patient technology literacy	0	0	0	0	0	0
14. Site staff adaptation						
15. Maintaining protocol adherence	0	0	0	0	0	

*11.What strategies have been most effective in overcoming the possible challenges with DCTs? (Please rank all the following using the arrow button or by dragging the choices,where1=the most effective strategy)

☐ 1. Partnering with experienced technology providers

☐ 2. Developing comprehensive staff & patient training

☐ 3. Early engagement with regulatory bodies

☐ 4. Implementing robust data management

☐ 5. Additional support for patient engagement

☐ 6. Conducting pilot studies before full implementation

5. PATIENTAND SITE PERSPECTIVE

*12.If you have collected any direct feedback, then how would you rate their overall satisfaction with DCT elements? (<u>If response to this question is Not Collected/Not Applicable then **skip to Q15**</u>)

	Very dissatisfied (1)	Dissatisfied (2)	Neither satisfied nor dissatisfied (3)	Satisfied (4)	Very satisfied (5)	Not Collected/not applicable
1. Patient satisfaction	0	0	0	0	0	0
2. Trial site staff satisfaction	0	0	0	0	0	0

13. What were the common positive aspects reported by the patients? (Please rank all The following, where1=the most common)

- ☐ 1. Convenience
- ☐ 2. Reduced travel time
- ☐ 3. Better integration with daily life
- ☐ 4. Improved communication with the site staffs
- ☐ 5. Access to real-time health data

14. What is your experience about the primary concerns of clinical trial sites regarding DCTs? (Please select all that apply)

1. Loss of patient engagement & relationships
2. Reduced site revenue
3. Technological challenges
4. Changes in staff roles & responsibilities
5. Data quality and integrity issues
6. Regulatory compliance uncertainties
7. Patient safety monitoring
8. Loss of control over trial processes
9. Concerns about long-term relevance of traditional site roles
10. Sites are not involved in the trial planning
11. Other (please specify)

6. FUTURE OUT LOOK AND INDUSTRY DEVELOPMENT

*15.Inthe next12months, what do you expect regarding your organization's use of the following?

	Decrease Significantly (1)	Decrease slightly (2)	Stay the same (3)	Increase slightly(4)	Increase significantly (5)
1. Full DCT					
2. Hybrid DCT					

*16.What do you see as the primary barriers to wider adoption of DCTs? (Please rank all the following, where1 = the biggest barrier)

- ☐ 1. Regulatory uncertainty
- ☐ 2. Lack of standardization across industry
- ☐ 3. Inability to measure returns on investment (ROI)
- ☐ 4. High implementation costs
- ☐ 5. Resistance to change (internal or external)
- ☐ 6. Technology limitations
- ☐ 7. Concerns about data quality/data privacy
- ☐ 8. Patient preference for traditional trials
- ☐ 9. Operational & Logistical Challenges
- ☐ 10. Site adoption

17. Please share any additional insights or experiences regarding DCTs that you feel are important.

18. For access to the survey results, enter your email below (optional):

I'll send you the report link when published. Your email won't be linked to your responses or shared with anyone except the survey author. This survey remains anonymous.

Email address [ ]

Thank you for your time spent taking this survey. Your response has been recorded.
