# Supplementary material for: Stakeholder-reported perspectives on decentralized clinical trials: A multi-country descriptive survey
Source: Contemp Clin Trials Commun. 2026 Sep 28;54:101694. doi: 10.1016/j.conctc.2026.101694 (PMC13657102; doi:10.1016/j.conctc.2026.101694)
Supplement: Supplementary file 2 [file mmc2.pdf]

Supporting Information file S2

Table 1. Country-Wise Response Distribution

S. No.	Names of the Country	Respondent counts
1	Algeria	1
2	Australia	5
3	Austria	3
4	Belgium	1
5	Brazil	2
6	Canada	4
7	Chile	1
8	China	1
9	Denmark	34
10	Egypt	2
11	France	8
12	Germany	3
13	Hungary	1
14	India	128
15	Ireland	4
16	Italy	4
17	Kenya	1
18	Korea	2
19	Latvia	1
20	Mexico	1
21	Netherlands	2

22	Nigeria	2
23	Norway	4
24	Pakistan	1
25	Poland	4
26	Portugal	1
27	Romania	1
28	Singapore	2
29	South Africa	2
30	Spain	2
31	Sweden	3
32	Switzerland	3
33	Taiwan	3
34	Thailand	2
35	Turkey	1
36	United Kingdom	10
37	United States	37
38	West Africa	1
Grand Total		288
